# Psychiatric Symptoms as the First Clinical Presentation of Multiple Sclerosis: A Case Report

**DOI:** 10.7759/cureus.1474

**Published:** 2017-07-15

**Authors:** Muhammad Aadil, Aitzaz Munir, Muhammad Jahanzaib Anwar, Hasnain Arshad, Ibrar Anjum, Aniqa Faraz

**Affiliations:** 1 Department of Medicine, FMH College of Medicine; 2 Department of Psychiatry, Howard University Hospital; 3 Department of Internal Medicine, Rush University Medical Center; 4 Department of Internal Medicine, Howard University Hospital; 5 Internal medicine, University of health science Lahore; 6 Department of Internal Medicine, King Edward Medical University Lahore, Pakistan

**Keywords:** neuropsychiatry, multiple sclerosis, aggressive behavior, mood disorder

## Abstract

Multiple sclerosis (MS) is an autoimmune condition which affects the axon myelination in the brain. There can be multiple ways it can present initially, but physical signs and symptoms are the most common ones. We are reporting a case of MS from Pakistan which presented with neuropsychiatric features and was offered psychiatric care, but the patient declined treatment because of stigma related to psychiatric care. Four months later when her condition worsened, further investigation showed it to be a case of MS. The purpose of this case report is that psychiatric features should be considered for differentials of MS.

## Introduction

Multiple sclerosis (MS) is a disease caused by an immune-mediated process against central nervous system (CNS) causing neural demyelination and degeneration resulting in interruption of nerve impulses traveling to and from the brain [1]. This inflammatory process causes progressive loss of neural functions causing sensory loss, spasticity, muscle cramping, pain, optic neuritis, transverse myelitis, trigeminal neuralgia, etc. which are common presentations of MS [2]. Currently, available therapies for MS are rarely effective to halt disease progression and cause undesirable side effects. It is one of the most common causes of neural disability in young adults, affecting about 2.3 million people worldwide in 2013 [3]. The current prevalence of this disease in the USA is 90 per 100,000 population [1]. However, the data is not available to confirm its prevalence rate in Pakistan. Multiple sclerosis can present as relapsing-remitting MS (most common 85% of cases), primary progressive, secondary progressive and progressive-relapsing [1-2]. Peculiar disease course of relapsing-remitting MS has always been the key to its diagnosis in the past. It very rarely presents with a psychiatric symptom as reported by Mahboobi in his case report, where delirium was the first symptom of MS, in 2014-15 [4].

In the current case, a young woman presented with inappropriate behavior suggestive of bipolar disorder as a presenting symptom of MS which was overlooked for a long time by the healthcare providers until she started showing signs of physical disability.

## Case presentation

**Figure 1:**
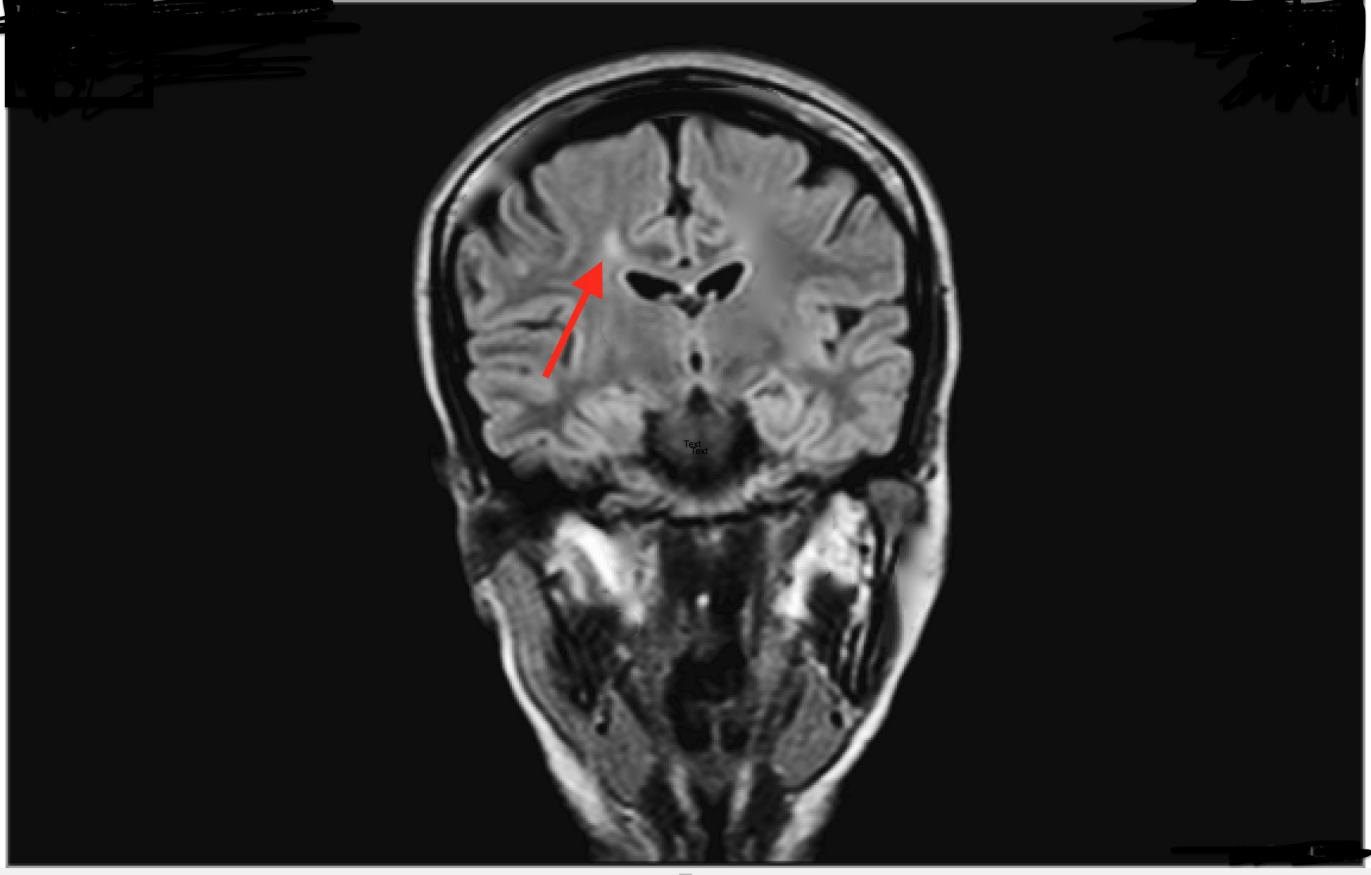
Hyperintense lesions at T2 weighted image (T2WI) and fluid-attenuated inversion recovery (FLAIR) sequence shown by arrow.

A 27-year-old Pakistani woman presented in outpatient department with complaints of spells of crying, laughing without any reason and aggressive behavior for three years with few period of being symptom-free. Her general physician considering her symptoms referred her to a psychiatrist. The psychiatrist keeping histrionic personality disorder as the top most differential diagnosis gave her some medication (which she didn’t have any record of) and offered some cognitive therapy. The patient's family stopped taking her to the session due to the stigma of psychiatric treatment. Her condition did not improve. Four months before presentation, she started to develop gradual, progressive and painful weakness of right lower limb. The next three months she could not do her daily routine activities and eventually became bedridden. On examination, she had bilaterally positive Babinski sign, increased tone in the lower extremity, exaggerated knee reflex, and diminished ankle reflex, power in lower limb was ⅖ and decreased sensation as well. In upper limbs, she had a normal tone with inverted biceps reflex and absent brachioradialis reflex, sensations were intact in the upper limb. There was decreased sensation on left side of the face suggesting left trigeminal nerve involvement. Taste sensation was lost on the right side of anterior two-third of the tongue indication right facial damage. Rest of the cranial nerves were normal in function. The fundoscopic examination was normal. A provisional diagnosis of inflammatory demyelinating diseases was made. Magnetic resonance imaging (MRI) of brain and spinal cord without gadolinium contrast were done which showed abnormal signal intensity areas involving multiple bilateral subcortical superficial white matter of frontal, parietal and temporal regions and deep white matter of right parietal and left temporal regions. Lesions were hypointense on T1 weight image (T1W1) and hyperintense on T2W1 and fluid-attenuated inversion recovery (FLAIR) sequence. There was no perilesional edema, mass effect or midline shift. MRI of spine did not show any abnormality. Cerebrospinal fluid (CSF) examination showed positive oligoclonal bands, and hence the patient was labeled as Primary progressive multiple sclerosis (Figure 1).

## Discussion

This case report highlights that behavioral changes can be the first presenting symptoms of MS. This presentation is very rare. We conducted a brief literature review search using Pubmed, Google Scholar, Scopus which yielded no similar presentation in Pakistan. To our knowledge, this is the first case reported from this region.

Claudia Diaz-Olavarrieta along with her co-authors conducted a study on patients with MS, and the results showed patients developing neuropsychiatric symptoms as the disease evolved. Depression was the most common manifestation in the studied patients (79%). The other psychiatric alterations these patients developed include, agitation 40%, anxiety 37%, irritability 35%, apathy 20%, euphoria 13%, disinhibition 13% and hallucinations 10% [5]. The pathophysiology of the development of these symptoms remains to be poorly understood. Studies indicate that demyelinating lesions and neural degeneration at the temporal lobe of the brain, which is the language and emotion center, can be the cause of these symptoms [6-7]. Fazzito, et al. reported multiple cases of MS with some psychiatric alterations. In these cases, three patients manifested psychosis as the disease progressed, only one presented with depression and one with changes in behavior as presenting complaints, respectively. She described these alterations being common during the disease course but only 1% of MS cases present with psychiatric symptoms as an initial symptom [8]. Psychiatric symptoms manifestation is prevalent in established MS, but initial presentation with these symptoms is rare. Studies showed that more than half of the patients with MS develop depression or another psychiatric symptom during the disease, overlooking these symptoms can result in underdiagnosis and complications [9]. In another case report, published in 2009 by Smith, a female patient presented with erotomanic delusion and complaints of sexual harassment. Her diagnosis of MS was later confirmed by MRI of the brain and lumbar puncture [10].

In our case, the patient presented with laughing and crying spells along with aggressive behavior. She was diagnosed with a psychiatric condition and was being treated. This resulted in progression of her underlying MS which caused her pain and suffering. When the patient developed signs of physical disability limiting her activities, the physician did further examination which showed signs suggestive of demyelinating disease. As her symptoms started three years ago, she was diagnosed with primary progressive MS. MRI of brain and spinal cord along with CSF examination confirmed the diagnosis of MS.

## Conclusions

Formal evaluation for any organic cause should be done if the patient presents with a psychiatric symptom unresponsive to therapy. An underlying cause should be kept in mind as misdiagnosis could result in poor prognosis and increased patient suffering. By diagnosing such patients early and providing them with proper treatment, we can reduce physical disability.

## References

[1] Hersh CM (2014). Multiple sclerosis. Multiple.

[2] Luzzio C (2017). Multiple sclerosis. Multiple.

[3] Moore S (2013). Major depression and multiple sclerosis - a case report. J Med Life.

[4] Mahboobi N, Nolden-Hoverath S, Rieker O (2015). Multiple sclerosis presenting as a delirium: a case report. Med Princ Pract.

[5] Politte LC, Huffman JC, Stern TA (2008). Neuropsychiatric manifestations of multiple sclerosis. Prim Care Companion J Clin Psychiatry.

[6] Gudiene D, Burba B (2003). Mental disorders and their relation to brain lesion location: diagnostic problems. Medicina (Kaunas).

[7] Reiss JP, Sam D, Sareen J (2006). Psychosis in multiple sclerosis associated with left temporal lobe lesions on serial MRI scans. J Clin Neurosci.

[8] Fazzito MM, Jordy SS, Tilbery CP (2009). Psychiatric disorders in multiple sclerosis patients. Arq Neuropsiquiatr.

[9] Jongen PJH (2006). Psychiatric onset of multiple sclerosis. J Neurol Sci.

[10] Smith EJ (2009). Multiple sclerosis presenting with erotomanic delusions in the context of "Don't ask, don't tell". Mil Med.

